# Effects of Viscosities and Solution Composition on Core-Sheath Electrospun Polycaprolactone(PCL) Nanoporous Microtubes

**DOI:** 10.3390/polym13213650

**Published:** 2021-10-23

**Authors:** Yan Chen, George Z. Tan, Yingge Zhou

**Affiliations:** 1 Systems Science and Industrial Engineering, Binghamton University, Binghamton, NY 13901, USA; ychen474@binghamton.edu; 2 Industrial, Manufacturing, and Systems Engineering, Texas Tech University, Lubbock, TX 79401, USA; george.z.tan@ttu.edu

**Keywords:** core-sheath electrospinning, nanoporous microtubes, tissue engineering, advanced manufacturing

## Abstract

Vascularization for tissue engineering applications has been challenging over the past decades. Numerous efforts have been made to fabricate artificial arteries and veins, while few focused on capillary vascularization. In this paper, core-sheath electrospinning was adopted to fabricate nanoporous microtubes that mimic the native capillaries. The results showed that both solution viscosity and polyethylene oxide (PEO) ratio in polycaprolactone (PCL) sheath solution had significant effects on microtube diameter. Adding PEO into PCL sheath solution is also beneficial to surface pore formation, although the effects of further increasing PEO showed mixed results in different viscosity groups. Our study showed that the high viscosity group with a PCL/PEO ratio of 3:1 resulted in the highest average microtube diameter (2.14 µm) and pore size (250 nm), which mimics the native human capillary size of 1–10 µm. Therefore, our microtubes show high potential in tissue vascularization of engineered scaffolds.

## 1. Introduction

Tissue engineering is a multi-disciplinary field that aims at repairing tissues and organs to restore, maintain, and improve biological functions [1,2,3]. One of the most important factors in tissue engineering is the fabrication of biomimetic scaffolds where various advanced manufacturing techniques were applied [4]. However, the lack of capillary vascularization has limited cell proliferation and infiltration in the scaffolds. To overcome this limitation, we adopted a novel core-sheath electrospinning technique to produce nanoporous microtubes that highly assembles native human capillaries.

Electrospinning has been a widely used technique that attracts numerous attentions owing to its flexibility, versatility, cost-efficiency, and operability [5,6,7,8]. It takes advantage of various materials to fabricate fibers with desired properties. Parameters such as voltage and pump rate can be altered to obtain desired experimental results. The electrospun polymers can also achieve considerable mechanical properties in the micro or nano scales [9]. Therefore, electrospinning techniques were applied in many areas, such as wound dressing [10], drug delivery [11], and biomanufacturing [12].

According to the spinneret type, the electrospinning process can be divided into needle-less, single, coaxial electrospinning [13]. Among them, coaxial is often used to produce core-sheath microfibers to obtain composite fibers or hollow structures [14]. For example, Duan and Greiner combined coaxial electrospinning with air-blowing assistance to electrospinning core-sheath fibers, metal-in-carbon fibers, and hollow fibers [12]. The range of flow rate was 5.8–12 mL/h, which is higher than conventional electrospinning and successfully produced core-sheath fibers on a large scale. In another case, Simões et al. loaded Nimesulide into PMMA-PCL (poly(methyl methacrylate)-polycaprolactone) core-sheath by coaxial electrospinning for drug delivery [9]. The results showed core-sheath structures achieve better Nimesulide delivery compared to pure PMMA fibers.

Previously, our group did a series of experiments to fabricate polylactic acid (PLA) nanoporous microtubes to mimic the fenestrated capillaries. Various material variables such as solvent volatility, solution viscosity, and solution composition were investigated to explore the effects on microtube diameter and nanopore size [2,8,14]. However, as one of the most widely used polymers in the biomedical and tissue engineering field, the fabrication of polycaprolactone (PCL) nanoporous microtubes has yet to be made.

In this paper, a novel core-sheath electrospinning technique was adopted to fabricate PCL nanoporous microtubes. The objective of this study is to investigate the effects of solution viscosity and sheath solution composition on microtubes’ outer diameter and pore size on the sheath. The hypotheses are (1) higher viscosity of core-sheath solution contributes to better tubular structure with higher diameter, (2) adding PEO into the PCL solution can increase the pore size on sheath due to rapid evaporation of the solvent and the dissolving of PEO. To test the hypotheses, three solution viscosity levels and three PCL/PEO ratios in sheath solution were selected. The results showed that both solution viscosities and PCL/PEO ratio in the sheath solution have significant effects on microtube diameter and pore size, although some of the effects were non-linear. This paper has the potential of contributing to future tissue vascularization applications as the nanoporous microtubes highly mimic human capillary vessels.

## 2. Materials and Methods

### 2.1. Polymer Solutions Preparation

Polycaprolactone (PCL, molecular weight = 80,000) pellets, polyethylene glycol (PEO, molecular weight = 100,000) power were purchased from Sigma-Aldrich (St. Louis, MI, USA). Dichloromethane (DCM) was purchased from Marron Fine Chemicals (Radnor, PA, USA). Deionized water (DI water) was obtained from a Millipore Milli-Q system.

Three levels PCL solutions (low: 7%, middle: 10%, and high: 15% w/v) and three levels of PEO solutions (low: 8%, middle: 12%, and high: 17.5% w/v) were prepared by dissolving PCL and PEO in DCM through magnetic stirring for 4 h at room temperature. Sheath solutions with different PCL/PEO ratios were prepared by adding PEO solution into PCL solution with respective volume ratios (1:0, 3:1, and 1:1).

### 2.2. Electrospinning Setting and Experiment Design

The electrospinning process was performed on the TL-Pro robotic electrospinning platform (Tong Li Tech, Shenzhen, China) with a 50 kV high voltage power source. A concentric core-sheath spinneret was adopted for this study. The PCL/PEO solution and PEO solution were pumped from a two-channel syringe pump with a 0.5 mL/h pump rate. The syringe pump of PCL/PEO mixed solution was connected to the sheath of the spinneret, and the pure PEO solution was connected to the core of the spinneret. The solution and parameter settings are summarized in Table 1. The nozzle size for core and sheath solution were gauge 25 and gauge 18, respectively. The tip-to-ground distance was set at 220 mm. The positive voltage ranged from 8.5 to 9 kV. The electrospinning time was around 20 min.

Based on previous research, we proposed that the solution viscosity levels influence the formation of enclosed hollow microtubes. Besides, we also hypothesized that the morphology of nanopores on the microtube would be different by adding different ratios of water-soluble PEO solution into the PCL sheath solution. Therefore, to investigate the effects of solution viscosity and the ratio of PEO in PCL solution, a two-factor full factorial experiment was designed as follows (Table 1). Three levels of viscosities (63–69 mPa·s, 237 mPa·s, and 1066–1077 mPa·s) and three levels of PCL to PEO ratios (by volume) in the sheath solution (1:0, 3:1, and 1:1) were chosen. Since the viscosity and composition are independent from each other, there was no interaction between the two factors. For the 3^2^ design experiment without interaction, a total of 9 groups of experiments were conducted.

### 2.3. Post-Processing of Microtubes

**Figure 1:**
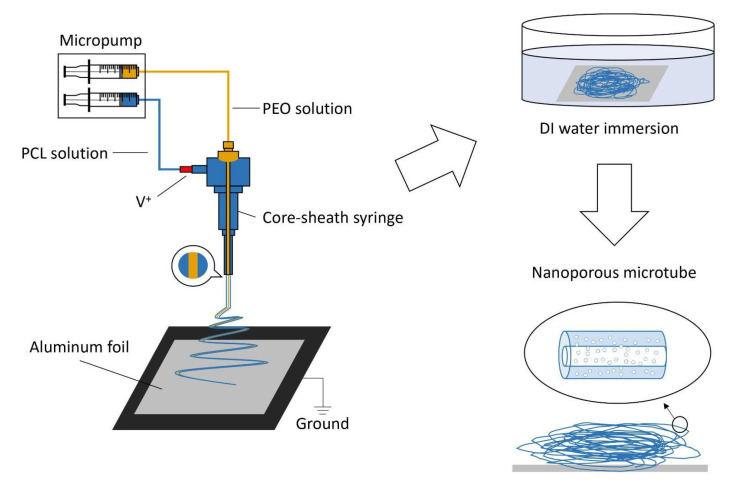
Schematic illustration of processing the electrospun.

A schematic illustration of processing the electrospun microtubes is shown in Figure 1. By adopting the coaxial spinneret, PCL/PEO and PEO solutions were electrospun simultaneously into microfibers with a core-sheath structure. The fabricated microfibers were collected by aluminum foil and immersed in deionized water overnight to dissolve the PEO. After the water bath and air-drying process at room temperature, the hollow structure was formed and the pores on the sheath also showed up.

### 2.4. Characterization of Microtubes

**Figure 2:**
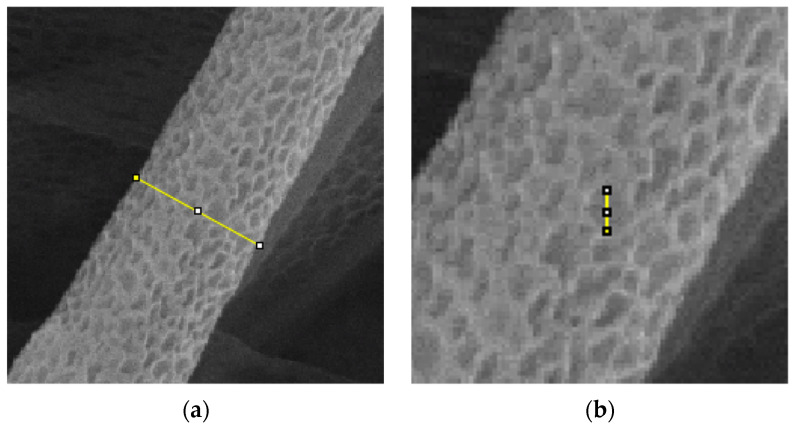
Measurement for (**a**) microtube diameter and (**b**) pore size.

The microtubes were coated by sputter coating for 1 min and examined under scanning electron microscopy (SEM, Zeiss, Jena, Germany). Thirty fibers and thirty pores were selected randomly from each sample. Fiber diameter and nanopore size were analyzed by ImageJ (National Institutes of Health, Bethesda, MD, USA). The measurement method is shown in Figure 2.

## 3. Results

### 3.1. Solution Viscosity Level

**Figure 3:**
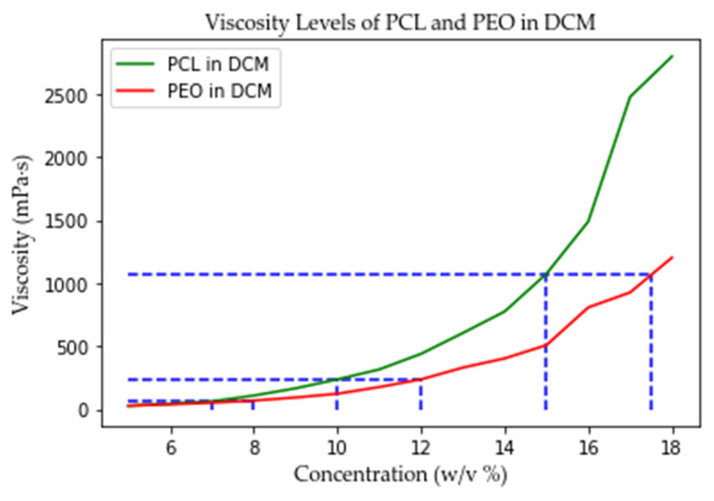
The viscosity of PCL and PEO.

The solution concentrations were selected according to the viscosity levels of PCL and PEO (Figure 3). The viscosity differences between same level concentration PCL and PEO solutions were negligible compared to different level solutions. The viscosities of the polymer solutions were measured by a digital rotational viscometer (IKA, Wilmington, NC, USA).

### 3.2. Characterization of Microtube Morphology

**Figure 4:**
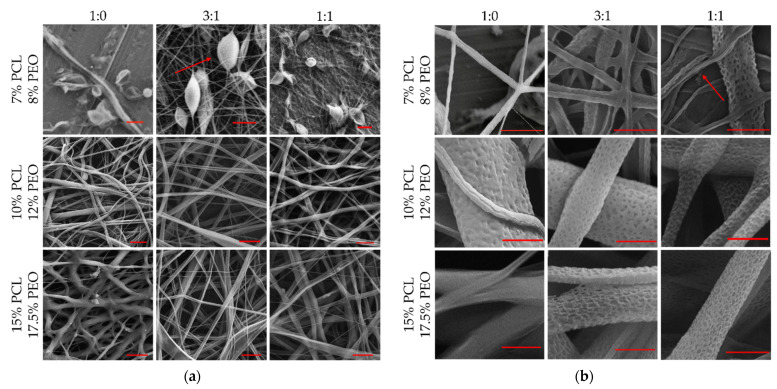
SEM images for PCL nanopores microtubes. (**a**) microtubes under 10 µm scale bar; (**b**) nanopores under 2 µm scale bar.

The SEM images of microfibers were taken after the overnight water bath and shown in Figure 4. Figure 4a shows the microtube structure and Figure 4b shows the nanopores geometry. All nine groups obtained microscale tubular structures. Groups 1–3 with lower solution viscosity levels shown less enclosed tubular structures (Figure 4b low viscosity) and more beads (Figure 4a low viscosity) in the sample. By increasing the concentration of core-sheath solutions, the viscosities of solutions also increased, and more enclosed tubes can be observed. Also, beads were eliminated by increasing solution concentration/viscosity. For middle and higher viscosity groups, adding PEO into the PCL sheath solution contributes to nanopores formation. The possible reason is that water-soluble PEO occupied some of the space in the microtube surface and was dissolved after the water bath, showing more nanopores on the microtubes. For Figure 4b, the sheath solution of the Groups 1, 4, and 7 are pure PCL solutions and only nonpenetrating dents were observed on the tube surface of Group 4 due to the high volatility of DCM solvent. We did not observe visible nanopores in Groups 1–3. On the other hand, Groups 5, 6, 8, and 9 showed deeper and larger nanopores on the microtube surface.

### 3.3. Fiber Diameter and Pore Size Analysis

The diameter and pore size data are listed in Table 2. Since no nanopores were identified on the microtubes in Groups 1–4, and 7, the pore sizes were not included. Therefore, only the groups with middle and high levels of concentration with PEO added were included in pore size data analysis. The average diameter for the nine groups ranged from submicron to micron size and the average pore size was in the submicron range. In all 9 groups, Group 2 has the lowest average microtube diameter and Group 8 has the highest average pore size.

**Figure 5:**
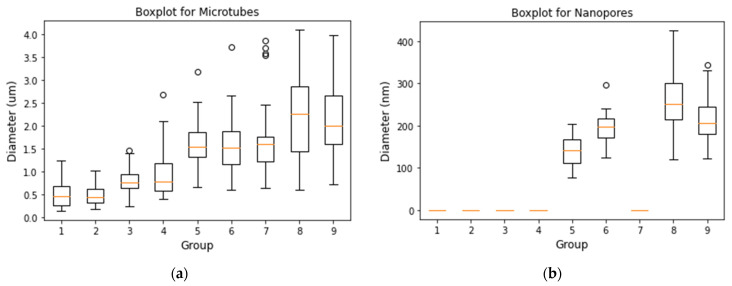
(**a**) Boxplot of microtubes’ and (**b**) nanopores’ diameter.

Figure 5a shows the boxplot of microtubes and Figure 5b shows the boxplot of nanopores. The microtubes ranged from 0.141~4.099 µm and the nanopores ranged from 77~425 nm. Overall, the microtube diameter is positively correlated to adding PEO solution into PCL sheath solution and increasing solution concentration viscosity. Effects of further increasing the PEO ratio in sheath solution showed various results depending on solution viscosities. This indicates that PCL/PEO ratio and solution viscosity plays interaction roles on microtube diameter. Similarly, effects of the PCL/PEO ratio on pore size showed opposite results when solution concentration changed from middle to higher levels (Figure 5b). This again indicates that PEO ratio and solution viscosity plays interaction roles in pore size.

**Figure 6:**
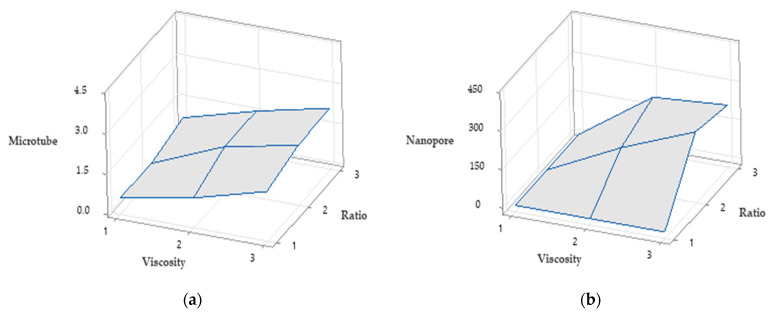
Surface change response of (**a**) microtubes and (**b**) nanopores.

Statistical analysis of microtube diameter and pore size was conducted by Minitab using a 3 × 3 design. The two factors are solution viscosity and PCL/PEO ratio in sheath solutions. The viscosity has three levels: low, middle, and high, and the ratios have three levels: 0, 3:1, and 1:1. Figure 6a shows the surface change response of microtube diameter with different viscosity and PCL/PEO ration, and Figure 6b shows the surface change response of nanopores. In these two plots, 1–3 in x axis stands for low, middle, and high three viscosity levels. 1–3 in y axis stands for 0, 3:1, and 1:1 three ratio levels. The z axis in Figure 6a shows the diameter of microtubes and the z axis in Figure 6b indicates the range of pore sizes.

**Figure 7:**
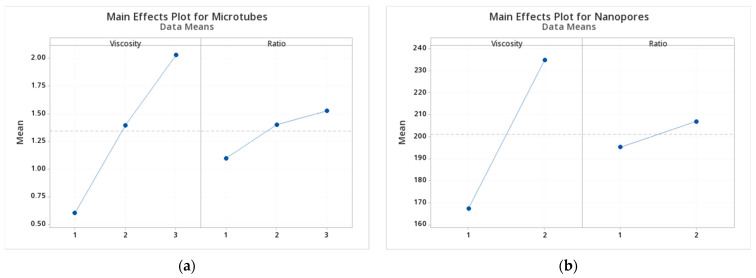
Main Effects Plot for Microtubes (**a**) and Nanopores (**b**).

From the ANOVA table (Table 3), the *p*-values of both viscosity and PCL/PEO ratio are smaller than 0.05, showing a significant influence on the microtube diameter. On the other hand, the interaction of viscosity and composition ratio showed a marginal significant influence on microtube diameter (*p*-value = 0.1 > 0.098 > 0.05). Figure 7 shows the main effect plots of viscosity and solution ratio on diameter and pore size. A larger range of variables stands for a higher influence on the result. Figure 7a suggests that viscosity has more effect on microtube diameter than PCL/PEO ratio.

As mentioned before, Groups 1–4 and 7 did not observe visible nanopores under SEM. Therefore, a 2 × 2 design was adopted for statistical analysis. The levels of viscosity are middle and high; the levels of PCL/PEO ratio are 3:1 and 1:1. As we can see from the ANOVA analysis in Table 4, the *p*-value of viscosity is 0.000 < 0.05, which shows a significant effect. While the *p*-value of the PCL/PEO ratio is 0.208 > 0.05 and did not significantly influence the pore size. Similarly, the interaction of Viscosity × Ratio is influenced by solution ratio and doesn’t show significant influence on pore size (*p*-value = 0.820 > 0.05). Figure 7b suggests that viscosity has more effect on pore size than PCL/PEO ratio.

## 4. Discussion

Various manufacturing approaches have been adopted to promote vascular network formation in engineered tissues, such as fused deposition modeling (FDM), selective laser sintering (SLS), micro-extrusion, and stereolithography (STL). FDM and SLS were used to create sacrificial geometries that were removed later to form channels [15]. For example, 3D printed polyvinyl alcohol (PVA) was dissolved in water and replaced with HepG2 cells within crosslinked gelatin to create a microvascular network [16]. Also, perfusable channels can be fabricated by SLS polycaprolactone (PCL) with sodium chloride as the porogen [17]. Micro-extrusion was introduced to deposit bioinks layer-by-layer. Endothelial cells with biocompatible hydrogels can be printed with intrinsic channels to create controllable flows [18]. However, the nozzle size limited extrusion-based bioprinting to fabricate features smaller than 50 μm. Therefore, it is not suitable for microvasculature fabrication.

Electrospinning showed great potential in vascularization for tissue engineering. For example, natural proteins and fibrins can be blended [19,20] or co-electrospun [21] with synthetic polymers to induce vascular formation or improve mechanical strength [22]. Duan et al. fabricated a tubular vascular tissue engineering scaffold with core-shell structured fibers with PCL as the core to providing mechanical support, and collagen as the shell to improve biocompatibility [23]. Also, the electrospun polymer can be modified physically or chemically to facilitate the vascularization process. For example, peptides modified poly(ester-urethane) urea (PEUU) was electrospun to improve endothelialization and prevent thrombi or hyperplasia [24]. Bolbasov sputter-coated a layer of thin titanium on electrospun scaffolds to enhance cell adhesion [25]. On the other hand, fiber geometry such as alignment can provide direction for cell spreading and guided neovascularization [26]. Our previous studies introduced a divergence electrospinning configuration to fabricate three-dimensional (3D) nanofiber scaffolds with gradient microstructures [27,28,29,30,31]. Human fibroblasts were cultured in the 3D scaffolds and the nanofibers guided cell orientation.

In this paper, we adopted a core-sheath electrospinning configuration to fabricate nanoporous microtubes which mimic human capillaries. Core-sheath electrospinning, or coaxial electrospinning, is a widely used method to fabricate multi-material nanofibers. It can generate a fiber with different layers, or a tubular structure if the core dissolves. Xia et al. [32] and Loscertales et al. [33] reported the pioneering works in producing nanotubes using co-axial electrospinning in 2004. Since then, coaxial electrospinning has attracted much attention and has been applied in various areas, including bioengineering and pharmaceutics [13,34]. Depending on the materials and electrospinning configurations, the outside diameters of the tubes can be up to several micrometers [35] or down to 100 nm [36]. Sufficient viscosity of the sheath solution is required to overcome the interfacial tension and form a stable Taylor cone during the electrospinning [13]. Some studies have suggested that the core solution can have a lower viscosity (a minimum viscosity) to keep it intact and continuous inside the sheath fluid [37,38]. The concentration of polymer solution is a dominating factor for viscosity. Generally, the fiber diameter increases as the solution concentration increases. In this study, the outside diameters of the tubes ranged from 0.14–0.41 µm. The size of these microtubes was close to that of native human fenestrated capillaries, which range from 1–10 µm. Therefore, the microtubes have potential to function as capillaries in engineered tissues.

In addition to a tubular structure, nanosized pores formed on the surface of the microtubes during the core-sheath electrospinning due to phase separation. The pore sizes ranged from 77 to 425 nm. These pores may enhance the mass transport between the tube walls, which is one of the important functions of capillary vessels. Studies showed the pore formation was due to the vapor-induced phase separation (VIPS) [39], induced by absorption of water vapor [40]. A different approach is to use the non-solvent induced phase separation (NIPS), which involves the addition of non-solvent with a higher boiling point than that of the good solvent in the polymer solution [41]. For example, Nguyen et al. fabricated porous microtubes by using a mixture consisting of a volatile good solvent and a non-volatile poor solvent in a 90/10 ratio at a humidity of greater than 70% [42]. They also found that the density of pore distribution was negatively correlated with the core solution feed because a larger volume of solution led to slower evaporating and drying processes of the solution jet, which prevented phase separation.

In this study, DCM was used for both core and sheath solutions due to its high volatility, which contributes to the surface nanopore formation. In addition, we mixed water-soluble PEO with PCL for sheath solution and investigated the effects of the PCL/PEO ratio on the nanopore formation. The PEO in the sheath solution significantly increased the pore depth but had little effect on the pore size. In addition, no pores were found on the microbeads or microtubes when the solution viscosity was low. This is probably because the vicinity of the sheath surface was saturated with DCM due to the high volatility of DCM and low PEO concentration. As a result, it was more difficult for water vapor to penetrate the DCM vapor and reach the solid-air interface to form pores [43]. When the sheath solution viscosity increased to 237 mPa·s (Groups 5, 6, 8, and 9), the addition of PEO facilitated the pore formation. This is consistent with Li et al.’s study that the pore size increased when adding water-soluble solvent into the solution [44]. However, when the PCL/PEO ratio increased from 3:1 to 1:1, the pore size increased when the sheath solution viscosity was 237 mPa·s, but the pore size decreased when the sheath solution viscosity was 1077 mPa·s. This mixed result is also consistent with other studies [44,45,46,47]. It showed that viscosity and PEO ratio have interactive effects on pore size.

## 5. Conclusions

In summary, this study investigated the effects of solution viscosity and composition in core-sheath electrospinning of nanoporous PCL microtubes. Our results suggest that the higher viscosity of core and sheath solutions contribute to higher microtube diameter and pore size. Additionally, adding PEO into PCL sheath solution is also beneficial to surface pore formation, although the effects of further increasing PEO showed mixed results in different viscosity groups. Microbeads were also showed up in lower viscosity groups, which is helpful for viscosity range selection in future studies. Our study showed that a high viscosity group with a PCL/PEO ratio of 3:1 resulted in the highest average microtube diameter (2.14 µm) and pore size (250 nm), which mimics the native human capillary size of 1–10 µm. These microtubes will be beneficial for angiogenesis and capillary formation in engineered scaffolds. Future work will focus on increasing the microtube diameter and pore size further to be highly aligned with capillary geometry, as well as fabrication of 3D aligned microtubes for future tissue engineering applications.

## Figures and Tables

**Table 1:** Materials for the core-sheath electrospinning.

Group Number	Sheath	Core	Viscosity (Sheath) (mPa·s)	Viscosity (Core) (mPa·s)
1	7% PCL ^1^	8% PEO ^1^	63	69
2	7% PCL/8% PEO (3:1)	8% PEO	63	69
3	7% PCL/8% PEO (1:1)	8% PEO	63	69
4	10% PCL	12% PEO	237	237
5	10% PCL/12% PEO (3:1)	12% PEO	237	237
6	10% PCL/12% PEO (1:1)	12% PEO	237	237
7	15% PCL	17.5% PEO	1077	1066
8	15% PCL/17.5% PEO (3:1)	17.5% PEO	1077	1066
9	15% PCL/17.5% PEO (1:1)	17.5% PEO	1077	1066

^1^ PCL = polycaprolactone; PEO = polyethylene glycol.

**Table 2:** Fiber diameter and pore size.

Group	Average Diameter (Tube)/µm	Standard Deviation (Tube)/µm	Average Diameter (Pore)/nm	Standard Deviation (Pore)/nm
1	0.517	0.309	-	-
2	0.472	0.201	-	-
3	0.824	0.274	-	-
4	1.023	0.597	-	-
5	1.593	0.513	138.533	34.653
6	1.575	0.611	196.467	34.961
7	1.764	0.836	-	-
8	2.141	0.884	252.033	68.442
9	2.183	0.824	217.567	53.553

**Table 3:** ANOVA table for microtube diameter.

Source	DF	Adj SS	Adj MS	F-Value	*p*-Value
Viscosity	2	91.782	45.891	117.060	0.000
Ratio	2	8.612	4.306	10.980	0.000
Viscosity × Ratio	4	3.062	0.7655	1.98	0.098

**Table 4:** ANOVA table for nanopores.

Source	DF	Adj SS	Adj MS	F-Value	*p*-Value
Viscosity	1	135879	135879	52.670	0.000
Ratio	1	4130	4130	1.600	0.208
Viscosity × Ratio	1	64033	64033	24.820	0.820

## Data Availability

The data presented in this study are available on request from the corresponding author.
